# Supplementary material for: Causal interplay between lactose intolerance and gut microbiota: a combined bidirectional Mendelian randomization and in vivo validation study
Source: Front Nutr. 2026 Jun 1;13:1803337. doi: 10.3389/fnut.2026.1803337 (PMC13265576; doi:10.3389/fnut.2026.1803337)
Supplement: Supplementary file 1 [file Data_Sheet_1.zip › supplementary materials/Forward/leave one out cross validation/ebi-a-GCST90027565.finngen_R12_E4_LACTONAS.pdf]

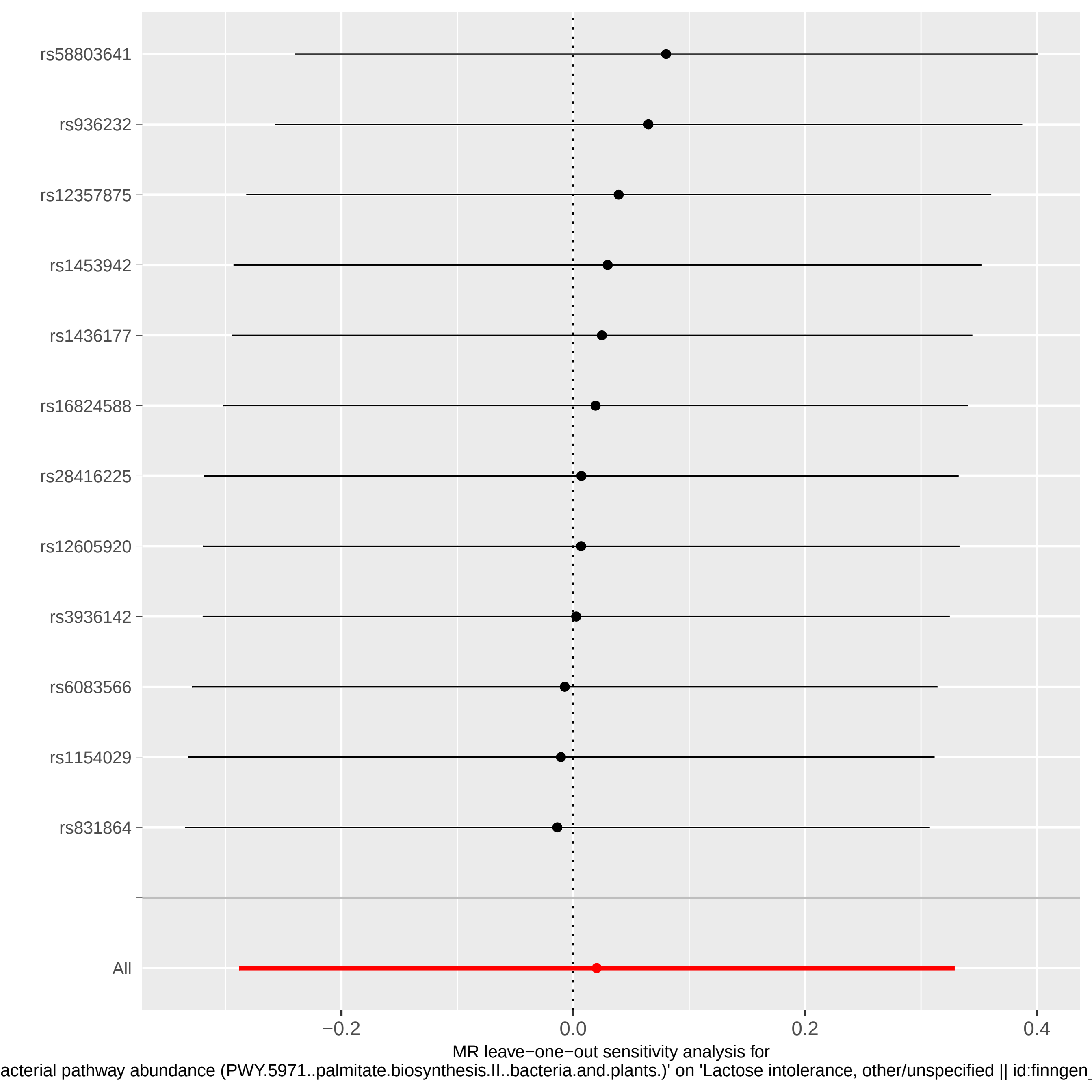
rs58803641
rs936232
rs12357875
rs1453942
rs1436177
rs16824588
rs28416225
rs12605920
rs3936142
rs6083566
rs1154029
rs831864
All
−0.2
0.0
0.2
0.4
MR leave−one−out sensitivity analysis for
acterial pathway abundance (PWY.5971..palmitate.biosynthesis.II..bacteria.and.plants.)' on 'Lactose intolerance, other/unspecified || id:finngen_
